# Supplementary material for: Deep representation learning of chemical-induced transcriptional profile for phenotype-based drug discovery
Source: Nat Commun. 2024 Jun 25;15:5378. doi: 10.1038/s41467-024-49620-3 (PMC11199551; doi:10.1038/s41467-024-49620-3)
Supplement: Supplementary file 1 — Supplementary Information [file 41467_2024_49620_MOESM1_ESM.pdf]

Supporting Information

Deep Representation Learning of Chemical-induced Transcriptional Profile for Phenotype-Based Drug Discovery

Xiaochu Tong[1,2], Ning Qu[1,2], Xiangtai Kong[1,2], Shengkun Ni[1,2], Jingyi Zhou[3,4,1], Kun Wang[5,1], Lehan Zhang[1,2], Yiming Wen[1,2,6], Jiangshan Shi[1,2], Sulin Zhang[1,2,*], Xutong Li[1,2,*]& Mingyue Zheng[1,2,6*]

*Correspondence: slzhang@simm.ac.cn; lixutong@simm.ac.cn; myzheng@simm.ac.cn;

[1]Drug Discovery and Design Center, State Key Laboratory of Drug Research, Shanghai Institute of Materia Medica, Chinese Academy of Sciences, 555 Zuchongzhi Road, Shanghai 201203, China

[2]University of Chinese Academy of Sciences, No. 19A Yuquan Road, Beijing 100049, China

[3]School of Physical Science and Technology, ShanghaiTech University, Shanghai 201210, China

[4]Lingang Laboratory, Shanghai 200031, China

[5]School of Life Sciences, Division of Life Sciences and Medicine, University of Science and Technology of China, Hefei 230026, China

[6]School of Pharmaceutical Science and Technology, Hangzhou Institute for Advanced Study, University of Chinese Academy of Sciences, Hangzhou 310024, China

Table of Contents:

Supplementary Fig. 1 Details of TranSiGen. S3

Supplementary Fig. 2 Distribution of Pearson's correlation coefficients of profiles for the same and random mechanism of action by TranSiGen-derived representation. S4

Supplementary Fig. 3 The Pearson's correlation coefficients within a group of active compounds and the Pearson's correlation coefficients between active and inactive compounds based on TranSiGen-derived representation. S5

Supplementary Fig. 4 The number of active compounds on each target from different cell lines. S6

Supplementary Fig. 5 Dimensionality reduction visualization of HTR2A active and inactive compounds based on various inferred perturbational representations. S7

Supplementary Fig. 6 Model performance of ligand-based virtual screening on target DRD2, ADRA2A, SLC6A4 and KCNH2. S8

Supplementary Fig. 7 Model performance of drug response prediction. S9

Supplementary Fig. 8 The max similarity of hit compounds screened by phenotype-based strategy and structural similarity-based strategy to the approved drugs. S10

Supplementary Table 1. The number of parameters for each model. S11

Supplementary Table 2. Model performance for inferring DEGs in chemical-blind setting at 5-fold cross validation (scenario 1). S12

Supplementary Table 3. Model performance for inferring DEGs in chemical-blind setting (scenario 1). S13

Supplementary Table 4. Model performance for inferring DEGs in cell-blind setting at leave-new-cell-out cross validation (scenario 2-1). S14

Supplementary Table 5. Model performance for inferring DEGs in cell-blind setting (scenario 2-2). S15

Supplementary Table 6. Model performance of ligand-based virtual screening on target HTR2A using different perturbational representations (chemical-blind). S16

Supplementary Table 7. Model performance of ligand-based virtual screening on target HTR2A using different perturbational representations (cell-blind). S17

Supplementary Table 8. Model performance of ligand-based virtual screening on target HTR2A within different thresholds of max similarity of test molecules relative to train data. S18

Supplementary Table 9. Details of drug response dataset collected from CTRP. S19

Supplementary Table 10. The screening performance of phenotype-based strategy and structural similarity-based strategy. S20

Supplementary Table 11. Details of the top 20 candidate compounds by TranSiGen_DISEASE screening. S21

Supplementary Table 12. The detailed prediction and experimental data for top 50 compounds screened by TranSiGen_DISEASE. S23

Supplementary Table 13. The detailed prediction and experimental data for top 50 compounds screened by TranSiGen_Drug. S26

Supplementary Figures

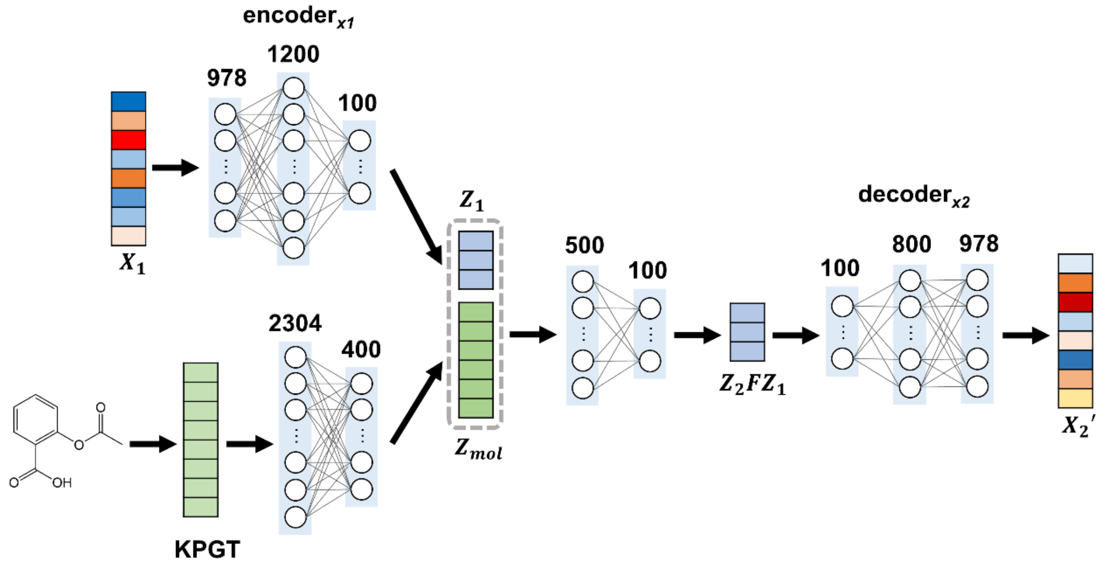

Supplementary Fig. 1 Details of TranSiGen. X_1 represents the control profile treated with DMSO, KPGT represents the 2034-dimensional molecular representation for the compound, Z_1 represents the latent representation of X_1, Z_{mol} represents the hidden representation of the compound, Z_2FZ_1 represents the latent representation from X_1 and perturbation representation, $X_2{}'$ represents the predicted transcriptional profile, $encoder_{x1}$ represents the encoder for X_1, and $decoder_{x2}$ represents the decoder for X_2.

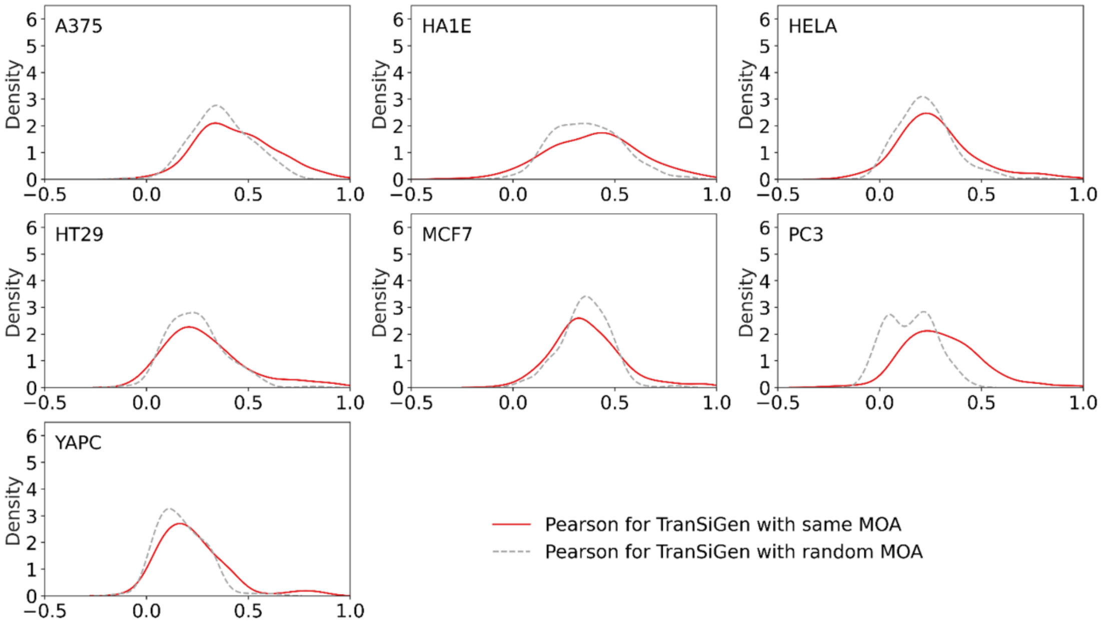

Supplementary Fig. 2 Distribution of Pearson's correlation coefficients of profiles for the same and random mechanism of action by TranSiGen-derived representation. Source data are provided as a Source Data file.

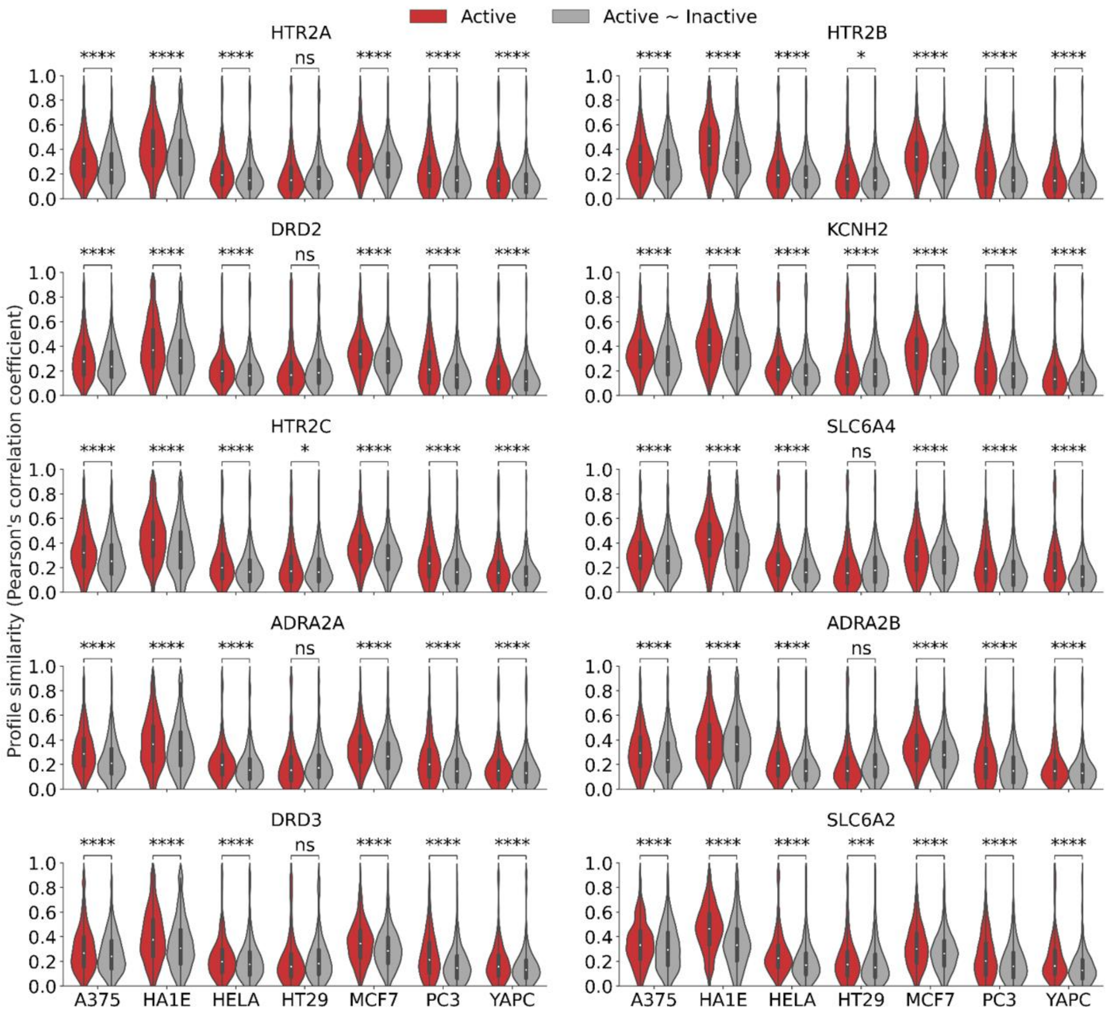

Supplementary Fig. 3 The Pearson's correlation coefficients within a group of active compounds and the Pearson's correlation coefficients between active and inactive compounds based on TranSiGen-derived representation. The black stick inside the violin displays the 25th and 75th percentiles of the data, the white dot indicates the median, and the boundaries of the violin plots refer to the maximum and minimum values, respectively. The one-sided Mann-Whitney test was used to analyze the data. The sample size for each test and the exact p-values are in source data. Source data are provided as a Source Data file. (Note: ****, $p < 0.0001$; ***, $0.0001 < p \leq 0.001$; **, $0.001 < p \leq 0.01$; *, $0.01 < p \leq 0.05$ and ns, $0.05 < p \leq 1.0$)

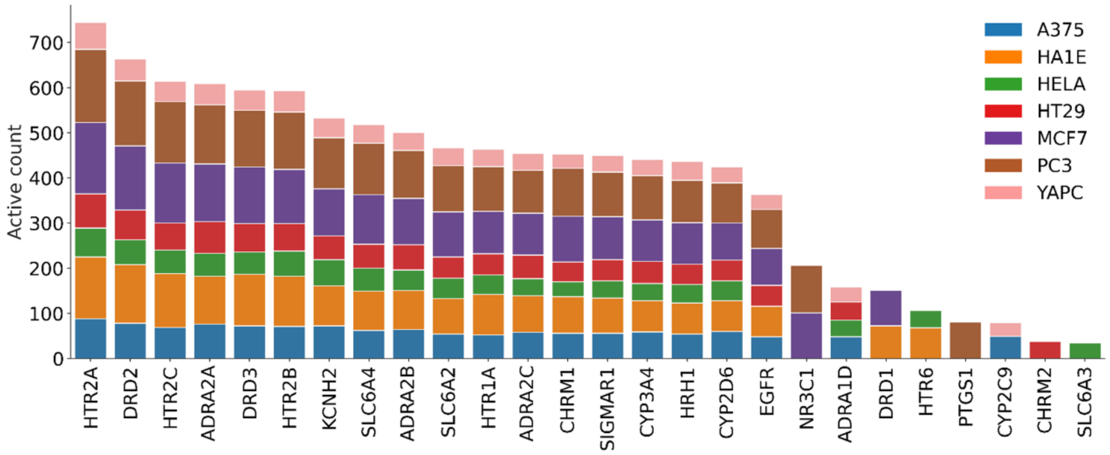

Supplementary Fig. 4 The number of active compounds on each target from different cell lines. Source data are provided as a Source Data file.

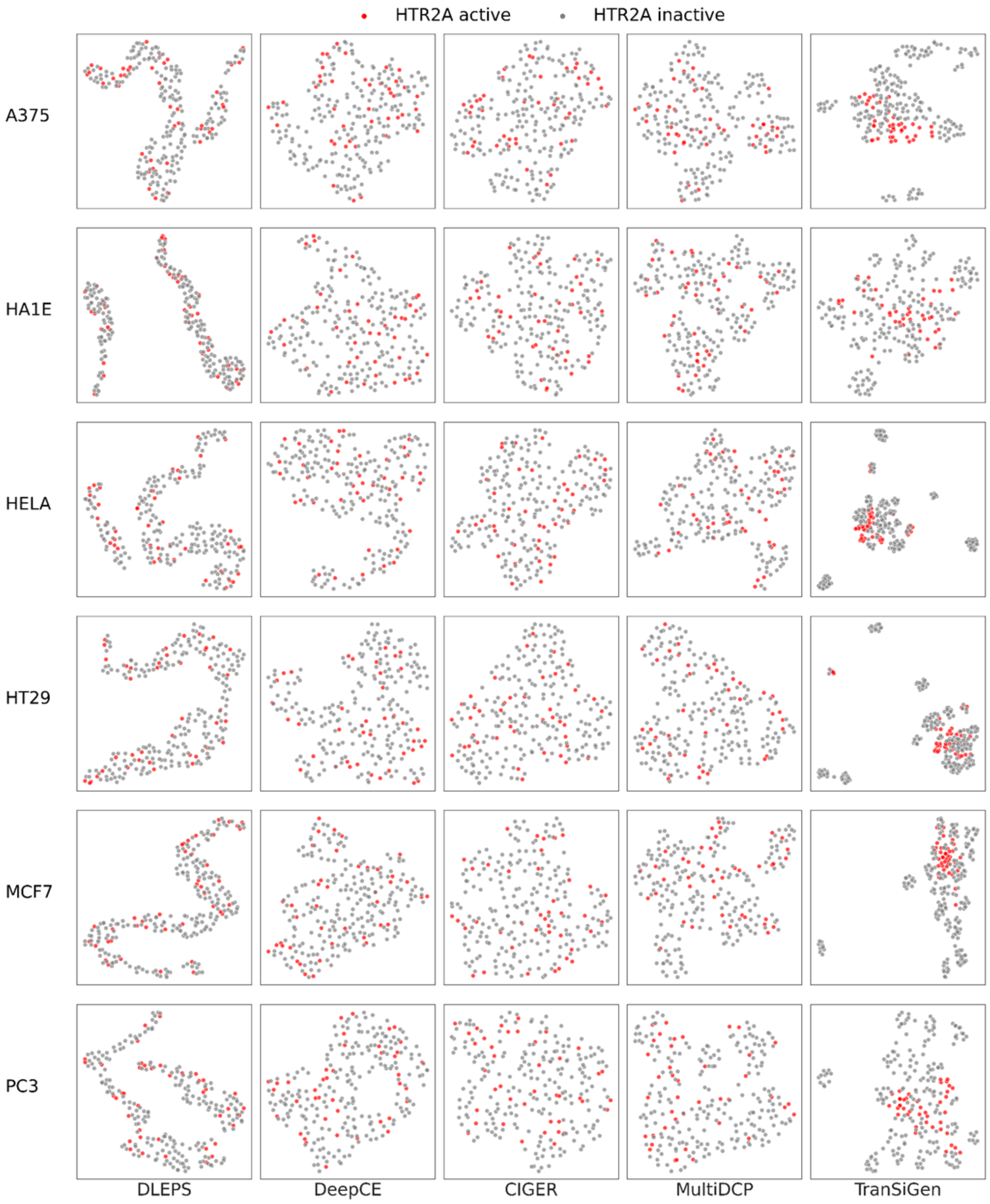

Supplementary Fig. 5 Dimensionality reduction visualization of HTR2A active and inactive compounds based on various inferred perturbational representations. Source data are provided as a Source Data file.

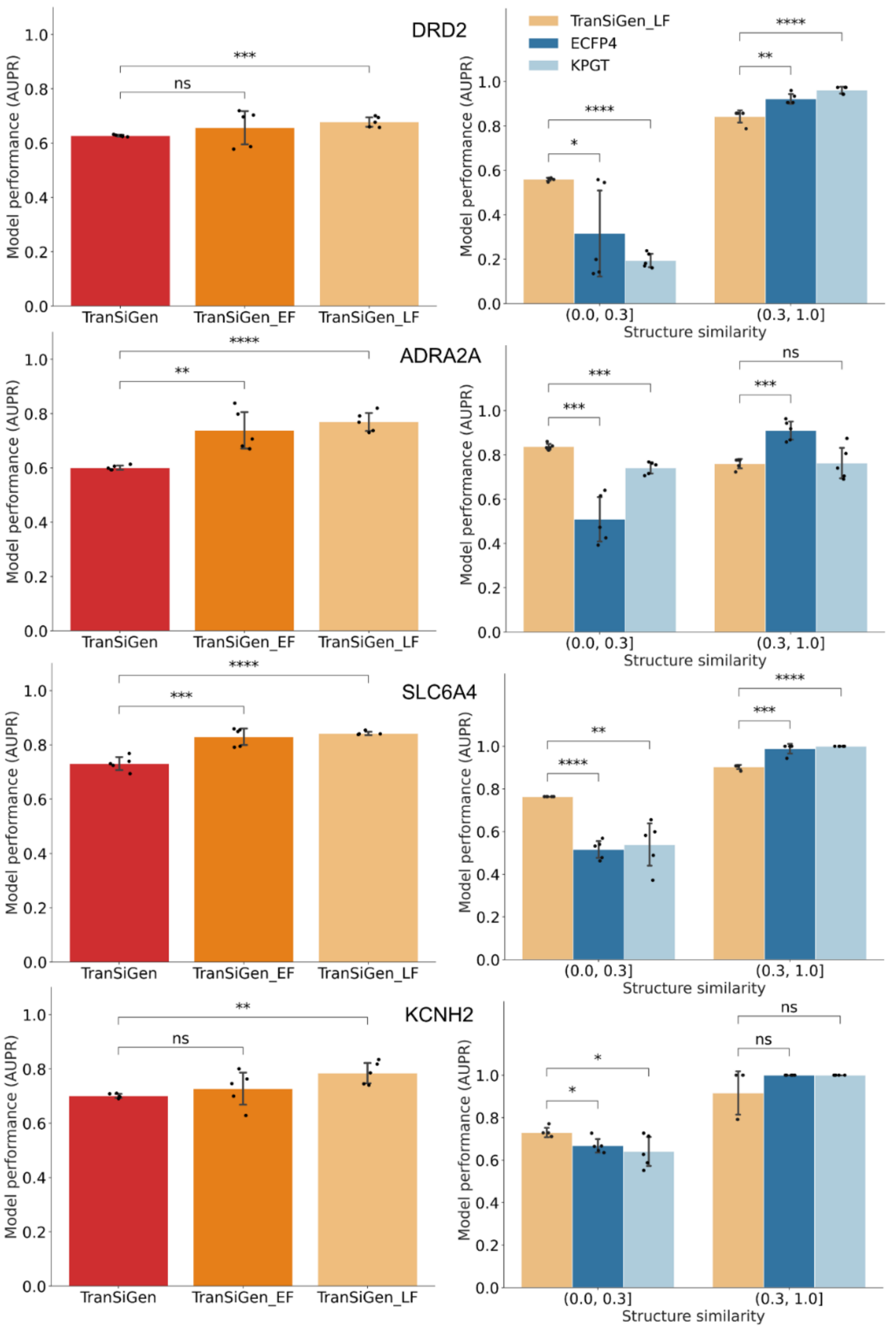

Supplementary Fig. 6 Model performance of ligand-based virtual screening on target DRD2, ADRA2A, SLC6A4 and KCNH2. All models were run five times with different random seeds. Black dots indicate the corresponding data points, and error bars represent the mean ± standard deviation. Two-sided t-test was applied between the models, and the exact p-values are in source data. Source data are provided as a Source Data file. (Note: ****, $p < 0.0001$; ***, $0.0001 < p \leq 0.001$; **, $0.001 < p \leq 0.01$; *, $0.01 < p \leq 0.05$ and ns, $0.05 < p \leq 1.0$)

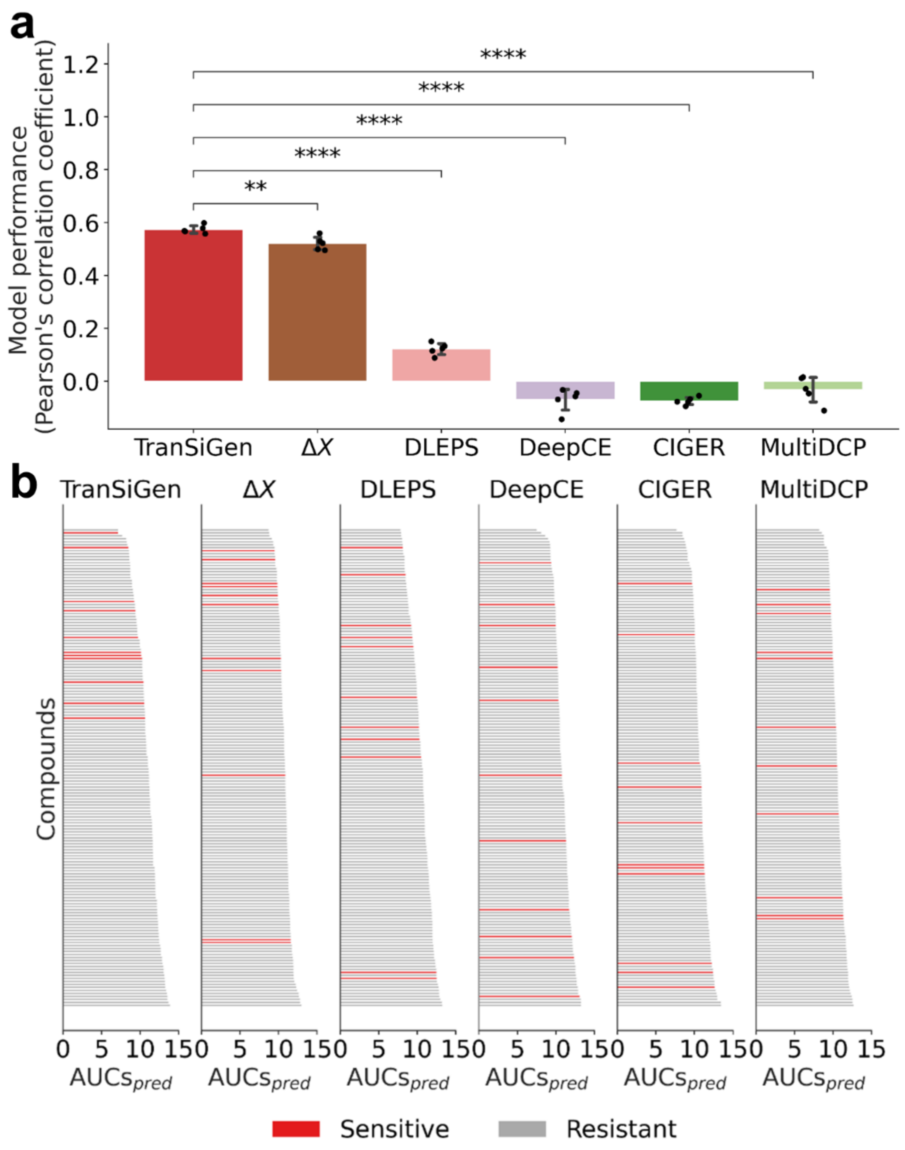

Supplementary Fig. 7 Model performance of drug response prediction. **a** Performance of predicting drug response using various type of representations. All models were run five times with different random seeds. Black dots indicate the corresponding data points, and error bars represent the mean ± standard deviation. Two-sided t-test was applied between the models, and the exact p-values are in source data. **b** Ranking results of compounds by AUCs_{pred} of models based on various type of representations. Source data are provided as a Source Data file. (Note: ****, $p < 0.0001$; ***, $0.0001 < p \leq 0.001$; **, $0.001 < p \leq 0.01$; *, $0.01 < p \leq 0.05$ and ns, $0.05 < p \leq 1.0$)

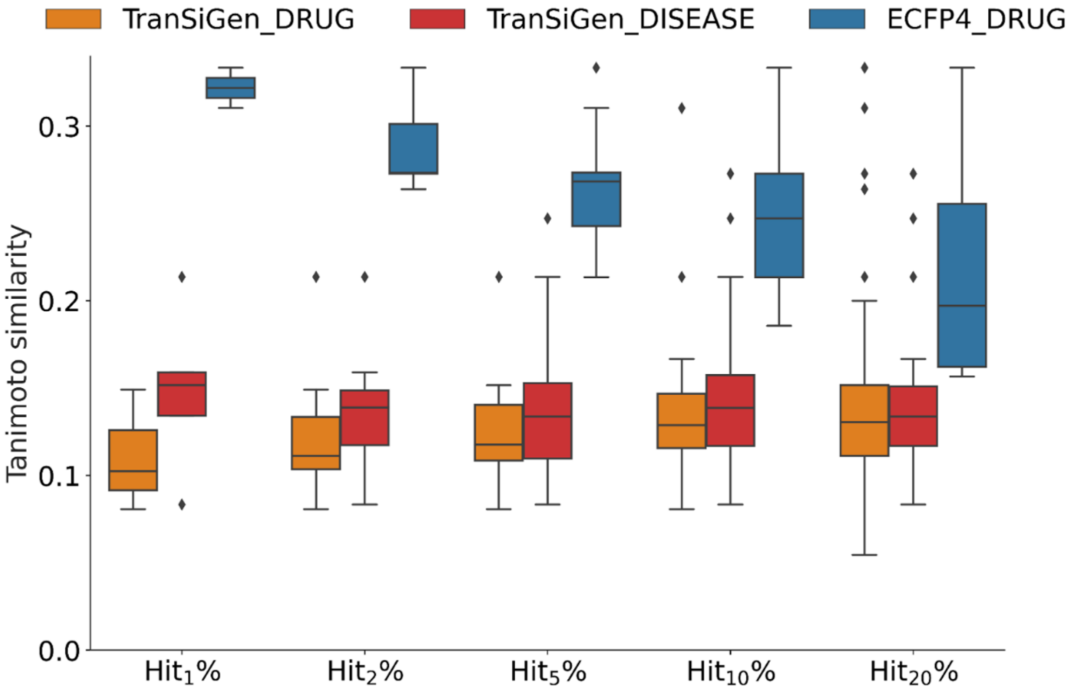

Supplementary Fig. 8 The maximum similarity of compounds at different hit rates (1%, 2%, 5%, 10%, and 20% of a total sample size of 1,625) was screened using both phenotype-based and structural similarity-based strategies, compared to three approved drugs. Box-and-whisker plots show the median (center line), 25th, and 75th percentile (lower and upper boundary), with 1.5× inter-quartile range indicated by whiskers, outliers shown as individual data points.

Supplementary Tables

Supplementary Table 1. The number of parameters for each model.

Model	#Params (MB)	Time (seconds)
DLEPS	20.441	0.021±0.003
DeepCE	2.605	0.017±0.004
CIGER	17.627	0.019±0.001
MultiDCP	4.317	0.035±0.013
TranSiGen	15.915	0.009±0.001

Supplementary Table 2. Model performance for inferring DEGs in chemical-blind setting at 5-fold cross validation (scenario 1).

Data	Model	RMSE	Pearson	Positive P@100	Negative P@100
seven cell lines (scenario 1-1)	DLEPS	1.495±0.040	0.427±0.019	0.246±0.013	0.309±0.012
	DeepCE	1.773±0.034	0.412±0.005	0.228±0.005	0.271±0.007
	CIGER	4.465±1.061	0.416±0.012	0.234±0.015	0.285±0.008
	MultiDCP	1.770±0.030	0.446±0.014	0.238±0.007	0.287±0.010
	TranSiGen (ECFP4)	0.690±0.022	0.533±0.016	0.379±0.012	0.394±0.012
	TranSiGen (KPGT)	**0.679±0.025**	**0.546±0.013**	**0.389±0.009**	**0.400±0.012**
full data (scenario 1-2)	TranSiGen (ECFP4; init_random)	0.526±0.005	0.606±0.004	0.433±0.005	0.442±0.001
	TranSiGen (ECFP4)	0.522±0.004	0.613±0.002	0.439±0.002	0.445±0.002
	TranSiGen (KPGT; init_random)	0.524±0.006	0.611±0.003	0.435±0.003	0.445±0.003
	TranSiGen (KPGT)	**0.518±0.003**	**0.617±0.002**	**0.443±0.002**	**0.450±0.001**

The bold font signifies the optimal performance.

Supplementary Table 3. Model performance for inferring DEGs in chemical-blind setting (scenario 1).

Data	Model	RMSE	Pearson	Positive P@100	Negative P@100	SSE	MSE	MAE	multiple r^2
seven cell lines (scenario 1-1)	DLEPS	1.551±0.005	0.418±0.004	0.244±0.002	0.316±0.002	2453.931±10.836	2.509±0.011	1.256±0.001	**0.155±0.004**
	DeepCE	1.773±0.034	0.43±0.02	0.238±0.003	0.29±0.01	3202.744±142.18	3.275±0.145	1.428±0.026	0.122±0.046
	CIGER	2.551±0.282	0.436±0.002	0.258±0.017	0.287±0.001	6647.359±1444.233	6.797±1.477	2.056±0.244	-1.016±0.464
	MultiDCP	1.782±0.022	0.457±0.004	0.240±0.011	0.302±0.002	3106.473±75.553	3.176±0.077	1.408±0.013	0.145±0.035
	TranSiGen (ECFP4)	0.661±0.003	0.517±0.001	0.363±0.002	0.387±0.003	470.711±4.38	0.481±0.004	0.489±0.004	-0.022±0.029
	TranSiGen (KPGT)	**0.641±0.002**	**0.54±0.002**	**0.381±0.002**	**0.397±0.001**	**446.824±2.102**	**0.457±0.002**	**0.474±0.001**	0.048±0.016
full data (scenario 1-2)	TranSiGen (ECFP4; init_random)	0.527±0.004	0.609±0.001	0.433±0.002	0.442±0.0	314.004±6.022	0.321±0.006	0.366±0.003	0.316±0.008
	TranSiGen (ECFP4)	0.522±0.005	0.615±0.002	0.441±0.0	0.448±0.004	309.535±6.284	0.316±0.006	0.362±0.004	0.328±0.004
	TranSiGen (KPGT; init_random)	0.524±0.004	0.613±0.001	0.437±0.002	0.445±0.002	310.245±5.429	0.317±0.006	0.364±0.002	0.322±0.005
	TranSiGen (KPGT)	**0.52±0.003**	**0.619±0.0**	**0.443±0.003**	**0.452±0.002**	**306.549±5.027**	**0.313±0.005**	**0.36±0.002**	**0.334±0.004**

The bold font signifies the optimal performance.

Supplementary Table 4. Model performance for inferring DEGs in cell-blind setting at leave-new-cell-out cross validation (scenario 2-1).

Model	RMSE	Pearson	Positive P@100	Negative P@100	SSE	MSE	MAE	multiple r^2
DeepCE	1.683±0.052	0.451±0.014	0.297±0.016	0.305±0.004	2895.239±175.707	2.960±0.180	1.366±0.062	**0.161±0.013**
CIGER	8.875±0.775	0.476±0.018	0.325±0.018	0.327±0.012	82027.450±15098.727	83.873±15.438	7.373±0.627	-26.709±5.303
MultiDCP	1.736±0.062	0.473±0.013	0.300±0.014	0.313±0.005	2950.825±208.212	3.017±0.213	1.369±0.058	0.129±0.042
TranSiGen	**1.040±0.034**	**0.486±0.023**	**0.333±0.019**	**0.382±0.021**	**1082.641±70.571**	**1.107±0.072**	**0.780±0.026**	-0.624±0.155

The bold font signifies the optimal performance.

Supplementary Table 5. Model performance for inferring DEGs in cell-blind setting (scenario 2-2).

Model	RMSE	Pearson	Positive P@100	Negative P@100	SSE	MSE	MAE	multiple r^2
TranSiGen (KPGT; 10 cells)	1.198±0.004	0.26±0.001	0.214±0.001	0.2±0.002	1455.064±9.649	1.488±0.01	0.884±0.003	-3.685±0.019
TranSiGen (KPGT; 50 cells)	1.073±0.008	0.297±0.003	0.241±0.002	0.223±0.004	1146.384±14.134	1.172±0.014	0.791±0.006	-2.761±0.006
TranSiGen (KPGT; 150 cells)	**0.96±0.012**	**0.324±0.003**	**0.256±0.002**	**0.232±0.004**	**917.258±23.053**	**0.938±0.024**	**0.701±0.013**	**-1.878±0.081**

The bold font signifies the optimal performance.

Supplementary Table 6. Model performance of ligand-based virtual screening on target HTR2A using different perturbational representations (chemical-blind).

Model	AUROC	AUPR	BACC	F1	Log_loss	MCC
DLEPS	0.566±0.000	0.265±0.000	0.583±0.000	0.329±0.000	0.688±0.000	0.164±0.000
DeepCE	0.527±0.041	0.225±0.030	0.557±0.041	0.315±0.074	0.635±0.097	0.102±0.066
CIGER	0.614±0.086	0.397±0.105	0.599±0.057	0.366±0.090	0.518±0.076	0.214±0.124
MultiDCP	0.547±0.069	0.267±0.062	0.558±0.045	0.283±0.095	0.613±0.133	0.115±0.090
TranSiGen	**0.889±0.041**	**0.731±0.092**	**0.784±0.047**	**0.620±0.076**	**0.369±0.022**	**0.523±0.103**

The bold font signifies the optimal performance.

Supplementary Table 7. Model performance of ligand-based virtual screening on target HTR2A using different perturbational representations (cell-blind).

Model	AUROC	AUPR	BACC	F1	Log_loss	MCC
DeepCE	0.556±0.059	0.238±0.014	0.569±0.038	0.318±0.065	0.535±0.015	0.124±0.054
CIGER	0.629±0.041	0.353±0.030	0.617±0.029	0.383±0.049	0.515±0.052	0.225±0.039
MultiDCP	0.488±0.006	0.229±0.004	0.522±0.004	0.212±0.013	0.705±0.138	0.061±0.025
TranSiGen	**0.855±0.037**	**0.710±0.062**	**0.805±0.017**	**0.681±0.031**	**0.369±0.021**	**0.602±0.040**

The bold font signifies the optimal performance.

Supplementary Table 8. Model performance of ligand-based virtual screening on target HTR2A within different thresholds of max similarity of test molecules relative to train data.

Structure similarity	Model	AUROC	AUPR	BACC	F1	Log_loss	MCC
(0.0, 0.3]	ECFP4	0.910±0.048	0.568±0.105	0.750±0.081	0.534±0.127	0.271±0.015	0.467±0.155
	KPGT	0.856±0.049	0.507±0.160	0.739±0.041	0.445±0.077	0.314±0.013	0.372±0.087
	TranSiGen (late fusion)	**0.977±0.010**	**0.856±0.056**	**0.846±0.010**	**0.700±0.046**	**0.261±0.003**	**0.657±0.053**
(0.3, 1.0]	ECFP4	0.938±0.014	0.852±0.063	0.847±0.054	0.800±0.075	0.416±0.027	0.719±0.052
	KPGT	**0.988±0.013**	**0.978±0.023**	**0.889±0.025**	**0.864±0.041**	**0.381±0.013**	**0.795±0.072**
	TranSiGen (late fusion)	0.955±0.024	0.921±0.043	0.845±0.024	0.801±0.032	0.543±0.007	0.700±0.062

The bold font signifies the optimal performance at the similarity thresholds (0.0, 0.3] and (0.3, 1.0].

Supplementary Table 9. Details of drug response dataset collected from CTRP.

Cell	Compound	AUC	AUC<5.5	AUC≥5.5
PC3	212	212	12	200
MCF7	208	208	21	184
A375	204	204	14	190
HT29	179	179	4	175

Supplementary Table 10. The screening performance of phenotype-based strategy and structural similarity-based strategy.

	$EF_{1\%}$	$EF_{2\%}$	$EF_{5\%}$	$EF_{10\%}$	$EF_{20\%}$
TranSiGen_DISEASE	**5.905**	**6.495**	**4.666**	**2.916**	1.977
TranSiGen_DRUG	3.543	4.133	3.732	2.799	**2.384**
ECFP4_DRUG	2.362	3.543	2.333	1.516	1.337

The bold font signifies the optimal performance.

Supplementary Table 11. Details of the top 20 candidate compounds by TranSiGen_DISEASE screening.

Rank	Name	Connectivity score	AUC in PRISM	Target	MOA
1	SB-939	-0.333	0.660	HDAC1, HDAC3, HDAC4, HDAC5, HDAC9, HDAC10	HDAC inhibitor
2	thiostrepton	-0.325		FOXM1	FOXM1 inhibitor, protein synthesis inhibitor
3	panobinostat	-0.323	0.728	HDAC1, HDAC2, HDAC3, HDAC4, HDAC6, HDAC7, HDAC8, HDAC9	HDAC inhibitor
4	belinostat	-0.319	0.874	HDAC1, HDAC2, HDAC3, HDAC4, HDAC5, HDAC6, HDAC7, HDAC8, HDAC9, HDAC10, HDAC11,	HDAC inhibitor
5	dacinostat	-0.288	0.834	HDAC1, HDAC2, HDAC3, HDAC4, HDAC5, HDAC6, HDAC7, HDAC8, HDAC9	HDAC inhibitor
6	BNTX	-0.287	0.823	OPRD1, OPRK1, OPRM1	opioid receptor antagonist
7	acetarsol	-0.272			
8	diroximel-fumarate	-0.267			anti-inflammatory agent
9	pelitinib	-0.262	0.850	EGFR	EGFR inhibitor
10	resibufogenin	-0.254			Na/K-ATPase inhibitor
11	neratinib	-0.252	0.754	EGFR, ERBB2, KDR	EGFR inhibitor
12	trichostatin-a	-0.252	0.574	HDAC1, HDAC2, HDAC3, HDAC4, HDAC5, HDAC6, HDAC7, HDAC8, HDAC9, HDAC10,	HDAC inhibitor
13	cyclovalone	-0.240	0.995	ABCG2	breast cancer resistance protein inhibitor
14	NSC-632839	-0.239	0.909	SENP2, USP1, USP2, USP7	ubiquitin specific protease inhibitor
15	LY2874455	-0.239	0.701	FGFR1, FGFR2, FGFR3, FGFR4, KDR	FGFR antagonist

16	pacritinib	-0.236	0.875	FLT3, JAK1, JAK2, JAK3	FLT3 inhibitor, JAK inhibitor
17	degarelix	-0.228		GNRHR	gonadotropin releasing factor hormone receptor antagonist
18	nexturastat-a	-0.227	1.348	HDAC1, HDAC6	HDAC inhibitor
19	rubitecan	-0.224	0.287	TOP1	topoisomerase inhibitor
20	resminostat	-0.222	0.944	HDAC1, HDAC3, HDAC6, HDAC8	HDAC inhibitor

Supplementary Table 12. The detailed prediction and experimental data for top 50 compounds screened by TranSiGen_DISEASE.

Rank	SMILES	Library	Cat. No.	Connectivity score	IC_{50}
1	C=C(NC(=O)c1csc(-c2nc3c(cc2O)-c2nc(cs2)C(=O)NC(C(C)O)C(=O)N/C(=C/C)c2nc(cs2)C(=O)NC2CC(O)C(=O)OCc4cccc5[nH]c(c(C)c45)C(=O)SCC(NC(=O)c4csc2n4)c2nc-3cs2)n1)C(N)=O	MedChemExpress	HY-107486	-0.374	> 50 µM
2	CN(c1ncccc1CNc1nc(Nc2ccc3c(c2)CC(=O)N3)ncc1C(F)(F)F)S(C)(=O)=O	MedChemExpress	HY-10459	-0.364	2.27 µM
3	Cc1cnc(Nc2ccc3c(c2)CCN(C(=O)/C=C/CN(C)C)C3)nc1-c1cnn(C(C)C)c1	MedChemExpress	HY-131906	-0.354	5.85 µM
4	O=C(Nc1c[nH]nc1-c1nc2cc(CN3CCOCC3)ccc2[nH]1)NC1CC1	MedChemExpress	HY-50514	-0.342	0.19 µM
5	CC(=O)NC1=NN(C(C)=O)C2(NC(=O)CS2)S1	Chemspace Library	CSCS00121601700	-0.336	> 50 µM
6	CC(C)CC(=O)Nc1n[nH]c2c1CN(C(=O)C1CCN(C)CC1)C2(C)C	MedChemExpress	HY-11001	-0.326	2.37 µM
7	CCOC(=O)/C(C#N)=C\c1ccc(N2C(=O)c3ccccc3C2=O)cc1	ChemDiv Library	3621-0032	-0.314	> 50 µM
8	CC[C@@]1(O)C(=O)OCc2c1cc1n(c2=O)Cc2cc3cc4c(cc3nc2-1)OCO4	MedChemExpress	HY-12486	-0.311	2.79 nM
9	Cc1[nH]c2ccccc2c1CCNCc1ccc(/C=C/C(=O)NO)cc1	MedChemExpress	HY-10224	-0.302	0.07 µM
10	CCCCc1nc2cc(/C=C/C(=O)NO)ccc2n1CCN(CC)CC	MedChemExpress	HY-13322	-0.301	1.95 µM
11	Cc1nnc(NC(=O)CCSCCC(=O)Nc2nnc(C)s2)s1	Chemspace Library	CSCS00048383301	-0.299	> 50 µM
12	O=C(/C=C/c1cccc(S(=O)(=O)Nc2ccccc2)c1)NO	MedChemExpress	HY-10225	-0.296	1.77 µM
13	Cc1nc(Nc2n[nH]c3c2CN(C(=O)N[C@H](CN(C)C)c2ccccc2)C3(C)C)c2sccc2n1	MedChemExpress	HY-13007	-0.293	2.48 µM
14	CCC(=O)CCCCC[C@@H]1NC(=O)[C@H]2CCCCN2C(=O)C([C@@H](C)CC)NC(=O)[C@H](Cc2cn(OC)c3ccccc23)NC1=O	MedChemExpress	HY-N6735	-0.291	1.44 µM
15	O=[Mo](=O)(O[Na])O[Na]	MedChemExpress	HY-D0851	-0.283	> 50 µM
16	COc1cc2c(cc1Nc1ncc(-c3cnn(C)c3)c(Nc3ccccc3P(C)(C)=O)n1)CN(C)CC2	MedChemExpress	HY-145107	-0.282	23.50 µM
17	Nc1ccccc1NC(=O)c1ccc(CNC(=O)OCc2cccnc2)cc1	MedChemExpress	HY-12163	-0.282	10.20 µM
18	O=C(/C=C/c1ccc(CN(CCO)CCc2c[nH]c3ccccc23)cc1)NO	MedChemExpress	HY-13606	-0.281	0.21 µM

19	C/C=C1\NC(=O)[C@H]2CSSCC/C=C/[C@H](CC(=O)N[C@H](C(C)C)C(=O)N2)OC(=O)C(C(C)C)NC1=O	MedChemExpress	HY-15149	-0.279	21.27 nM
20	N[C@H]1CC[C@H](Nc2nc(Nc3ccc(CN4CCOCC4)cc3)c3ncn(C4CCCC4)c3n2)CC1	MedChemExpress	HY-112145	-0.278	0.91 μM
21	COCC(=O)N1CCC(N(C)Cc2cccnc2)CC1C(=O)O	Ambinter	Amb22181211	-0.277	> 50 μM
22	CC(C=CC(=O)NO)=CC(C)C(=O)c1ccc(N(C)C)cc1	APEXBIO	A8183	-0.277	0.51 μM
23	Cn1ncc([N+](=O)[O-])c1C(=O)Nc1nc2ccccc2n1CCN1CCOCC1	Chemspace Library	CSCS00133001156	-0.275	> 50 μM
24	C=C(NC(=O)C(=C)NC(=O)c1csc(C2=N[C@@H]3c4csc(n4)[C@H]4NC(=O)c5csc(n5)[C@H]([C@](C)(O)[C@@H](C)O)NC(=O)[C@H]5CSC(=N5)/C(=C/C)NC(=O)C([C@@H](C)O)NC(=O)c5csc(n5)[C@]3(CC2)NC(=O)[C@H](C)NC(=O)C(=C)NC(=O)[C@H](C)NC(=O)C([C@@H](C)CC)NC2C=Cc3c([C@H](C)O)cc(nc3C2O)C(=O)O[C@@H]4C)n1)C(N)=O	MedChemExpress	HY-B0990	-0.275	5.02 μM
25	Cc1ccc(Cn2c(CNC(=O)c3cc([N+](=O)[O-])ccc3Cl)nc3cccnc32)cc1	ChemDiv Library	G831-0079	-0.275	> 50 μM
26	CO[C@@H]1CC[C@H]2[C@H]1OCCN2C(=O)c1cccc2c1OCCO2	Ambinter	Amb22884668	-0.275	> 50 μM
27	CO[C@@H]1[C@H](N(C)C(=O)c2ccccc2)C[C@H]2O[C@]1(C)n1c3ccccc3c3c4c(c5c6ccccc6n2c5c31)C(=O)NC4	MedChemExpress	HY-10230	-0.274	0.94 μM
28	CC(=O)N1CCc2ccccc2[C@H]1C(=O)NCc1ccc2c(c1)OCO2	ChemDiv Library	P368-0185	-0.271	> 50 μM
29	O=S(=O)(NCc1ccncc1)c1ccc(F)c(F)c1	MedChemExpress	HY-148043	-0.270	13.24 μM
30	C=C(NC(=O)C(=C)NC(=O)c1csc(C2=N[C@H]3c4csc(n4)[C@H]4NC(=O)c5csc(n5)[C@@H]([C@](C)(O)[C@H](C)O)NC(=O)[C@@H]5CSC(=N5)/C(=C\C)NC(=O)[C@H]([C@H](C)O)NC(=O)c5csc(n5)[C@]3(CC2)NC(=O)[C@@H](C)NC(=O)C(=C)NC(=O)[C@@H](C)NC(=O)[C@@H]([C@@H](C)CC)N[C@H]2C=Cc3c([C@@H](C)O)cc(nc3[C@@H]2O)C(=O)O[C@@H]4C)n1)C(N)=O	MedChemExpress	HY-B0990	-0.269	6.59 μM
31	O=C(Cc1nc(-c2ccccn2)n[nH]1)NCc1cccnc1	ChemDiv Library	8020-1328	-0.268	> 50 μM
32	CN1CCN(C(=O)N(CCCCCCC(=O)NO)c2ccc(-c3ccc4cnn(C)c4c3)cc2)CC1	MedChemExpress	HY-109109	-0.268	0.22 μM
33	CCN(CC)CCNC=C1C(=O)NC(=O)N(c2ccccc2C)C1=O	ChemDiv Library	3448-7402	-0.267	> 50 μM

34	CCn1nc(C)cc1C(=O)Nc1nc2cc(C(N)=O)ccc2n1C/C=C/Cn1c(NC(=O)c2cc(C)nn2CC)nc2cc(C(N)=O)cc(OCCCO)c21	MedChemExpress	HY-103665A	-0.266	> 50 μM
35	CC(/C=C/C(=O)NO)=C\[C@@H](C)C(=O)c1ccc(N(C)C)cc1	MedChemExpress	HY-15144	-0.264	0.59 μM
36	CN1CCc2c(c3ccccc3n2Cc2ccc(C(=O)NO)cc2)C1	MedChemExpress	HY-13271A	-0.264	28.20 μM
37	Cc1c(CC(=O)O)c(=O)oc2cc(N)ccc12	MedChemExpress	HY-D0025	-0.264	> 50 μM
38	COc1ccc(C[C@H](N)C(=O)N[C@H]2[C@@H](CO)O[C@@H](n3cnc4c(N(C)C)ncnc43)[C@H]2O)cc1	MedChemExpress	HY-B1743A	-0.263	> 50 μM
39	Nc1ccccc1NC(=O)c1ccc(CNc2nccc(-c3cccnc3)n2)cc1	MedChemExpress	HY-12164	-0.261	6.68 μM
40	COc1ccc(-c2n[nH]c(C)c2CC(=O)NCc2nc3ccccc3[nH]2)cc1	ChemDiv Library	Y042-6983	-0.259	> 50 μM
41	Cc1cc(=O)oc2cc(O)cc(O)c12	MedChemExpress	HY-N4102	-0.258	> 50 μM
42	CS(=O)(=O)N(Cc1nc(-c2cccs2)no1)Cc1ccccc1F	Chemspace Library	CSCS00132816410	-0.256	> 50 μM
43	CCOC(=O)[C@@]1(C)C=C(Nc2cccc(C(=O)O)c2)C(=O)N1c1cccc(C(=O)O)c1	ChemDiv Library	5340-0005	-0.256	> 50 μM
44	O=C(NC1CCNCC1)[C@@H]1CC[C@@H]2CN1C(=O)N2OS(=O)(=O)O	MedChemExpress	HY-16752	-0.255	> 50 μM
45	CN(C)Cc1c(C(=O)NCCOc2ccc(C(=O)NO)cc2)oc2ccccc12	MedChemExpress	HY-10990	-0.254	0.53 μM
46	Cc1nc2ncccc2c(=O)n1C1CN(C(=O)OC(C)(C)C)C1	Life Chemicals	F6668-2963	-0.254	> 50 μM
47	CC(C)(C)OC(=O)N1CCC(CC(=O)NCCOCCn2cccn2)CC1	Life Chemicals	F6573-2910	-0.253	> 50 μM
48	c1cnn(C(CNc2ncnc3cnccc23)c2ccsc2)c1	Life Chemicals	F6724-5302	-0.252	> 50 μM
49	C=CC(=O)N1CC2(CC(n3nc(-c4ccc5c(cnn5C)c4)c(-c4c(Cl)c(C)cc5[nH]ncc45)c3C)C2)C1	MedChemExpress	HY-139612	-0.251	11.00 μM
50	CNC(=O)c1cc2ccccc2[nH]1	Chemspace Library	CSSB00000206196	-0.251	> 50 μM

Supplementary Table 13. The detailed prediction and experimental data for top 50 compounds screened by TranSiGen_DRUG.

Rank	SMILES	Library	Cat. No.	Connectivity score	IC_{50}
1	Clc1ccc(C(c2ccc(Cl)cc2)[n+]2ccn(CC(OCc3ccc(Cl)cc3Cl)c3ccc(Cl)cc3Cl)c2)cc1	MedChemExpress	HY-103319	0.391	2.55 μM
2	Cc1c2ccncc2c(C)c2c1[nH]c1ccccc12	MedChemExpress	HY-15753	0.380	12.01 μM
3	COc1ccc(N(C(=O)CCl)C(C(=O)NCCc2ccccc2)c2cccs2)cc1	ChemDiv Library	K784-3188	0.357	0.12 μM
4	CCCCC(CC)CNC(=N)NC(=N)NCCCCCCNC(=N)NC(=N)NCC(CC)CCCC	MedChemExpress	HY-108547	0.353	1.18 μM
5	CCCCCCCCCCCCCCCC[N+](C)(C)CCN(Cc1ccc(OC)cc1)c1ncccn1	MedChemExpress	HY-B1246	0.351	2.55 μM
6	CCN(CC)CCN(Cc1ccc(-c2ccc(C(F)(F)F)cc2)cc1)C(=O)Cn1c(SCc2ccc(F)cc2)nc(=O)c2c1CCC2	MedChemExpress	HY-10521	0.351	3.76 μM
7	C[n+]1cn(CCCCCCn2cc(C(O)c3ccccc3)[n+](C)c2)cc1C(O)c1cccccc1	Chemspace Library	CSCS00160770112	0.349	> 50 μM
8	c1ccc2c(-c3cnn4cc(-c5ccc(N6CCNCC6)cc5)cnc34)ccnc2c1	MedChemExpress	HY-12071A	0.345	0.86 μM
9	COc1ccc(N(C(=O)CCl)C(C(=O)NCCc2ccccc2)c2cccs2)cc1Cl	MedChemExpress	HY-100002	0.331	40.67 nM
10	CCCCCCCCCCCCOc1c2cc(C[n+]3ccn(C)c3)cc1Cc1cc(C[n+]3ccn(C)c3)cc(c1OCCCCCCCCCCCC)Cc1cc(C[n+]3ccn(C)c3)cc(c1OCCCCCCCCCCCC)Cc1cc(C[n+]3ccn(C)c3)cc(c1OCCCCCCCCCCCC)C2	Life Chemicals	F1170-0374	0.330	28.68 μM
11	Nc1nc(NC[C@H]2CC[C@H](CNS(=O)(=O)c3cccc4ccccc34)CC2)nc2ccccc12	MedChemExpress	HY-107723	0.328	2.84 μM
12	COc1ccc(C(OC[C@H]2O[C@@H](n3ccc(NC(C)=O)nc3=O)[C@H](O[Si](C)(C)C(C)(C)C)[C@@H]2O)(c2ccccc2)c2ccc(OC)cc2)cc1	MedChemExpress	HY-138614	0.327	13.84 μM
13	O=C(CCl)N[C@@H](Cc1ccccc1)C(=O)N1C/C(=C\c2ccc([N+](=O)[O-])cc2)C(=O)/C(=C/c2ccc([N+](=O)[O-])cc2)C1	MedChemExpress	HY-136563	0.323	14.95 nM
14	C[C@H]1C[C@@H](C)CN(S(=O)(=O)c2ccc3c(c2)C(=NO)c2cc(S(=O)(=O)N4C[C@@H](C)C[C@@H](C)C4)ccc2C3=NO)C1	MedChemExpress	HY-109103	0.319	64.04 nM
15	CCCCCCC[n+]1ccc(-c2cc[n+](CCCCCCC)cc2)cc1	MedChemExpress	HY-101237	0.318	2.97 μM

16	O=C(Nc1cc(Cl)cc(Cl)c1O)c1c(O)c(Cl)cc(Cl)c1Cl	MedChemExpress	HY-17594	0.316	> 50 μM
17	O=S(=O)(c1ccc2c(c1)C(=NO)c1cc(S(=O)(=O)N3CCCCC3)ccc1-2)N1CCCCC1	MedChemExpress	HY-112063	0.313	1.05 μM
18	CC(C)(C)c1cc(C=C(C#N)C#N)cc(C(C)(C)C)c1O	MedChemExpress	HY-15511	0.306	1.00 μM
19	COc1cc(NS(C)(=O)=O)ccc1Nc1c2ccccc2nc2ccccc12	MedChemExpress	HY-13551	0.306	0.32 μM
20	Cc1cc2c(C(C)C)c(O)c(O)c(C=O)c2c(O)c1-c1c(C)cc2c(C(C)C)c(O)c(O)c(C=O)c2c1O	MedChemExpress	HY-13407	0.303	5.82 μM
21	Cn1nc(-c2ccccc2)nc2c(=O)n(C)c(=O)nc1-2	MedChemExpress	HY-125759	0.301	0.67 μM
22	CC(C)C[C@H](NC(=O)[C@H](CCc1ccccc1)NC(=O)CN1CCOCC1)C(=O)N[C@@H](Cc1ccccc1)C(=O)N[C@@H](CC(C)C)C(=O)[C@@]1(C)CO1	MedChemExpress	HY-10455	0.300	3.82 nM
23	c1cc(-c2cnn3cc(-c4ccc(N5CCNCC5)cc4)cnc23)c2cccnc2c1	MedChemExpress	HY-15897	0.298	1.59 μM
24	NC(=O)NN=C1c2cc(S(=O)(=O)N3CCCCCC3)cc([N+](=O)[O-])c2-c2c1cc(S(=O)(=O)N1CCCCCC1)cc2[N+](=O)[O-]	Life Chemicals	F1345-0152	0.296	> 50 μM
25	Cc1cc2c(c(O)c1-c1c(C)cc(O)c3c1C(=O)C=CC3=O)C(=O)C=CC2=O	MedChemExpress	HY-N3488	0.293	16.45 μM
26	O=C1/C(=C/c2ccc([N+](=O)[O-])cc2)CNC/C1=C\c1ccc([N+](=O)[O-])cc1	MedChemExpress	HY-136528	0.293	0.32 μM
27	CC(C)(O)C#Cc1ccc(C(=O)N2CCN(C(=O)c3ccc(C#CC(C)(C)O)cc3)CC2)cc1	ChemDiv Library	3472-2274	0.292	> 50 μM
28	CCCCCCCCCCCCCCCCn1cc[n+](Cc2c(Cl)cccc2Cl)c1C	MedChemExpress	HY-148189	0.291	0.04 μM
29	c1ccc(-c2ccc(CNc3nc(-c4ccccn4)nnc3-c3ccccc3)cc2)cc1	MedChemExpress	HY-12754	0.286	0.25 μM
30	CCCCCCCCCCCCn1cc[n+](C)c1	MedChemExpress	HY-W099544	0.286	0.08 μM
31	CCC1(O)C(=O)OCc2c1cc1n(c2=O)Cc2c-1nc1ccccc1c2CCNC(C)C	MedChemExpress	HY-13566A	0.286	5.68 nM
32	O=S(=O)(NC1CCCCC1)c1ccc2c(c1)C(=NO)c1cc(S(=O)(=O)NC3CCCCC3)ccc1-2	MedChemExpress	HY-103087	0.285	0.53 μM
33	Oc1c(Cl)cc(Cl)c(Cl)c1Cc1c(O)c(Cl)cc(Cl)c1Cl	MedChemExpress	HY-12637	0.284	8.83 μM
34	Cn1nc(-c2ccc(C(F)(F)F)cc2)nc2c(=O)n(C)c(=O)nc1-2	MedChemExpress	HY-18219	0.279	5.97 μM
35	O=C(Nc1ccc([N+](=O)[O-])c(Cl)c1)c1cc(Cl)ccc1O	MedChemExpress	HY-144770	0.277	2.24 μM
36	O=C(Nc1ccc([N+](=O)[O-])cc1Cl)c1cc(Cl)ccc1O	MedChemExpress	HY-B0497	0.273	0.76 μM

37	CCC1C/C(=C\c2cccnc2)C(=O)/C(=C/c2cccnc2)C1	MedChemExpress	HY-19625	0.273	0.61 μM
38	Oc1c(O)c(Cl)c(Cl)c(Cl)c1Cl	MedChemExpress	HY-W006000	0.272	51.81 μM
39	CCCCCCCCCCCCCCCCn1cc[n+](Cc2ccccc2)c1C	MedChemExpress	HY-100576	0.270	65.12 nM
40	CCOc1cc2ncc(C#N)c(Nc3ccc(OCc4cccc(F)c4)c(Cl)c3)c2cc1NC(=O)/C=C/CN(C)C	MedChemExpress	HY-103443	0.270	10.77 μM
41	CC(C)[C@H](CNC(=O)[C@@H](NC(=O)c1cccc(-c2ccccc2)n1)[C@@H](C)O)B(O)O	MedChemExpress	HY-10454	0.266	17.40 nM
42	CCCCCCCCNC(=N)NC(=N)NCc1ccc(Cl)c(Cl)c1	MedChemExpress	HY-125654A	0.264	3.84 μM
43	C/C=C(/C)C(=O)O[C@H]1C(C)=C2[C@@H]3OC(=O)[C@@](C)(O)[C@@]3(O)[C@@H](OC(=O)CCC)C[C@](C)(OC(C)=O)[C@H]2[C@@H]1OC(=O)CCCCCCC	MedChemExpress	HY-13433	0.264	9.61 nM
44	Cn1ncnc2c(=O)n(C)c(=O)nc1-2	MedChemExpress	HY-100760	0.263	35.48 nM
45	CC(C)C[C@H](NC(=O)[C@H](CCc1ccccc1)NC(=O)CO)C(=O)N[C@@H](Cc1ccccc1)C(=O)N[C@@H](CC(C)C)C(=O)[C@@]1(C)CO1	MedChemExpress	HY-150226	0.263	6.66 nM
46	O=C1/C(=C/c2ccccc2F)CNC/C1=C\c1ccccc1F	MedChemExpress	HY-119272	0.262	0.74 μM
47	Cc1nc2c(=O)n(C)c(=O)nc-2n(C)n1	MedChemExpress	HY-111117	0.261	0.62 μM
48	CC1(C)C=C(CN2C/C(=C\c3ccc(F)cc3)C(=O)/C(=C/c3ccc(F)cc3)C2)C(C)(C)N1O	MedChemExpress	HY-100453	0.260	0.88 μM
49	COc1ccc(C(NC(=O)CCc2ccccc2)c2ccc3cccnc3c2O)cc1	MedChemExpress	HY-147029	0.258	8.27 μM
50	O=C(O)/C=C/c1ccc(-c2ccc(O)c(C34CC5CC(CC(C5)C3)C4)c2)cc1	MedChemExpress	HY-14808	0.256	0.18 μM
